# Supplementary material for: Anti-Symmetric Electromagnetic Interactions’ Response in Electron Circular Dichroism and Chiral Origin of Periodic, Complementary Twisted Angle in Twisted Bilayer Graphene
Source: Molecules. 2022 Oct 2;27(19):6525. doi: 10.3390/molecules27196525 (PMC9572769; doi:10.3390/molecules27196525)
Supplement: Supplementary file 1 [file molecules-27-06525-s001.zip › molecules-1941149-supplementary.pdf]

molecules

Supporting Information

Anti-symmetric electromagnetic interactions Response in Electron circular dichroism and Chiral origin of Periodic complementary twisted angle in Twisted Bilayer Graphene

Guoqiang Dai [1,†], Xiangtao Chen [2,†], Ying Jing [1,*] and Jingang Wang [1,*]

1 College of Science, Liaoning Petrochemical University, Fushun 113001, China
2 School of Physics, Northeast Normal University, Changchun 130024, China
* Correspondence: jinying@lnpu.edu.cn (Y.J.); jingang_wang@lnpu.edu.cn (J.W.)
†: Equal contribution.

Table S1. The wavelength and oscillator intensity of main one-photon absorption excited state in Z-TwBLG-QDs-27.8° and Z-TwBLG-QDs-32.2°

Molecule	Excited states	Wavelength[nm]	Oscillator Strength
Z-TwBLG-QDs-27.8°	S_{15}	772.3	5.03
	S_{49}	612.8	0.31

Citation: Dai, G.; Chen, X.; Jing, Y.; Wang, J. Anti-Symmetric Electromagnetic Interactions' Response in Electron Circular Dichroism and Chiral Origin of Periodic, Complementary Twisted Angle in Twisted Bilayer Graphene. *Molecules* **2022**, *27*, 6525. https://doi.org/10.3390/molecules27196525

Academic Editors: Tian Wang, Cheng Zhong, Tianyuan Zhang and Xiaohua Wang

Received: 13 September 2022
Accepted: 27 September 2022
Published: 2 October 2022

Publisher's Note: MDPI stays neutral with regard to jurisdictional claims in published maps and institutional affiliations.

Copyright: © 2022 by the authors. Licensee MDPI, Basel, Switzerland. This article is an open access article distributed under the terms and conditions of the Creative Commons Attribution (CC BY) license (https://creativecommons.org/licenses/by/4.0/).

	S_{53}	598.1	1.30
Z-TwBLG-QDs-32.2°	S_{15}	772.4	5.03
	S_{49}	612.8	0.31
	S_{53}	598.2	1.29

Table S2. The wavelength and rotatory intensity of main ECD excited states in Z-TwBLG-QDs-27.8° and Z-TwBLG-QDs-32.2°

Twisted Angle	Excited states	Transition dipole moment			Transition magnetic dipole moment			Sm	Sr
		x	y	z	x	y	z		
27.8°	S_{15}	-4.2	-10.8	-6.2×10^{-4}	-0.0356	0.0195	-1.3×10^{-3}	0.870	0.971
	S_{49}	-2.1	-1.7	6.7×10^{-5}	0.0143	0.0183	-3.3×10^{-4}	0.602	0.880
	S_{53}	3.4	3.9	1.2×10^{-3}	-0.0251	-0.0703	-3.1×10^{-3}	0.607	0.880
32.2°	S_{15}	7.1	9.2	-3.2×10^{-4}	0.0117	-0.0212	4.0×10^{-4}	0.870	0.972
	S_{16}	-9.2	7.1	2.1×10^{-4}	0.0206	0.00947	-8.8×10^{-4}	0.870	0.972
	S_{49}	2.0	1.8	5.2×10^{-5}	-0.00879	0.0169	-4.9×10^{-4}	0.605	0.881
	S_{53}	3.2	4.0	1.8×10^{-4}	0.0301	-0.0530	2.6×10^{-3}	0.607	0.880

Table S3. The transition index of main one-photon excited states in Z-TwBLG-QDs-27.8° and Z-TwBLG-QDs-32.2°

Twisted Angle	Excited states	RMSD of hole in X Y Z			RMSD of electron in x y z			difference between RMSD of hole and electron		
		x	y	z	x	y	z	x	y	z
27.8	S_{15}	8.527	8.981	4.015	8.811	9.114	4.055	0.284	0.133	-0.002
	S_{49}	8.527	8.981	4.056	8.811	9.114	4.055	0.284	0.133	-0.002
	S_{53}	9.188	8.856	4.056	8.962	8.534	4.054	-0.226	-0.323	-0.002
32.2°	S_{15}	8.732	8.803	4.056	8.781	8.781	4.055	0.049	-0.025	-0.001
	S_{16}	8.803	8.732	4.056	8.779	8.779	4.055	0.025	0.049	-0.001
	S_{49}	8.654	8.872	4.056	8.892	9.037	4.054	0.239	0.165	-0.002
	S_{53}	9.260	8.785	4.056	9.025	8.443	4.054	-0.235	-0.342	-0.002

Twisted Angle	Excited states	∇_σ	H			H index	t	HDI	EDI
			x	y	z				
27.8°	S_{15}	0.015	8.669	9.047	4.056	13.170	-7.397	1.76	1.79

	S_{49}	0.270	8.773	8.955	4.055	13.176	-8.541	1.58	1.90
	S_{53}	-0.368	9.075	8.695	4.055	13.206	-4.955	1.91	1.70
32.2°	S_{15}	0.016	8.756	8.791	4.055	13.054	-8.750	1.75	1.78
	S_{16}	0.016	8.791	8.756	4.055	13.054	-8.763	1.75	1.78
	S_{49}	0.270	8.773	8.955	4.055	13.176	-8.541	1.58	1.90
	S_{53}	-0.387	9.143	8.614	4.055	13.200	-8.603	1.91	1.69

To deeply analyze the electronic transition behavior of Z-TwBLG-QDs, the transition density matrix (TDM) and electron-hole density of the main excited states are drawn and their transition index which is calculated in Table S3. The real space form $T(r;r')$ of the TDM between the ground state and the excited state is:

$$T(r;r') \equiv T(r_1;r_1') = \int \Phi^0(x_1,x_2,\ldots x_N)d\sigma_1 dx_2 dx_3\ldots dc_N \quad \text{(S1)}$$

where Φ^0 is the wavefunction of ground state， Ψ^{exc} is the wavefunction of excited state ， x is the spin space coordinate of the electron, σ is the spin coordinate, r is the space coordinate. The excited state of the system is calculated by using time-dependent density functional theory (TDDFT). The excited state wavefunction is described by the linear combination of various single excitation configuration functions. The $T(r;r')$ can be written more accurately as:

$$(r;r) = \sum_i \sum_a w_i^a \varphi_i(r)\varphi_a(r') \quad \text{(S2)}$$

where a and i are the numbers of empty orbitals and occupied orbitals, respectively, and w is the coefficient of the configuration function. Since r contains three components, $T(r;r')$ is a six-dimensional function. Take the diagonal element of TDM, let $r = r'$, then $T(r;r')$ becomes a three-dimensional function form $T(r)$:

$$(r;r) = \sum_i \sum_a w_i^a \varphi_i(r)\varphi_a(r) \quad \text{(S3)}$$

Then the $T(r)$ can be used to investigate the excitation characteristics from the ground state to the excited state by drawing.

Figure S1a indicates the TDM of Z-TwBLG-QDs-27.8° at S_{15}. The main diagonal represents the excitation characteristics of each layer of graphene, and the bright areas on both sides represent the charge transfer between layers. TDM shows that the transition density is mainly distributed around the diagonal, which belongs to the local excitation between layers. To quantitatively investigate the characteristics of local excitation, it is also necessary to perform wavefunction analysis to obtain the transition index of the excited state. The main excited state transition index is shown in Table S3, where S_r is used to characterize the overlap degree of electron and hole which increases as the value whose range is 0 to 1 increase. The S_r of S_{15} is close to 0.870, which indicates that the overlap of electron and hole distribution to a large extent; RMSD stands for the distribution breadth of holes and electrons, and the numerical value shows that the degree of dispersion of holes and electrons in the three components of x, y and z is similar, thus S_{15} is a typical state of local excitation. The electron-hole pair density shows that the hole-electron isosurface equably distributed on the edges of the top and underlying graphene (The red isosurface represents the place where the electron density increases, and the green represents the place where the hole decreases). Therefore, this local excitation is mainly contributed by the excitation of π electron. And the internal hole-electron isosurface centrosymmetric distributed on the Moiré, see Figure S1e, which indicates that π-π excitation dominated by edge of graphene plays an important role in the electron transition behavior of Z-TwBLG-QDs. As shown in Figure S1(b and f) and Figure S2(a

and d), when the twisted angle changes to 32.2°, the degenerated excited states S_{15} and S_{16} appear near 772nm, the transition index of degenerated excited states show that there are symmetric opposite dispersion degrees on the x and y components. And their t indexs are smaller than Z-TwBLG-QDs-27.8 °. The extension of holes and electrons are further reduced. However, the overlap degree of hole-electron and the distribution breadth are similar. Therefore, the excitation characteristics are similar, and they are all belong to local excitations.

The absorption peak of Z-TwBLG-QDs-27.8° at 612.8nm is contributed by S_{49}, the value of S_m (The physical meaning is similar to S_r) is 0.6, which Indicates that the degree of overlap of electrons and holes is reduced, compared with the excitation characteristics of 772nm, the difference of overall distribution breadth of hole-electron becomes larger. The transition density of the top graphene corresponds to the atom list in the lower left corner of the TDM, the transition density of the underlying graphene corresponds to the atom list in the upper right corner of the TDM, and there are two areas with obvious brightness on TDM, see Figure S1c. Combined with the electron-hole pair density, the basis function of the bright area corresponds to the area where the electron-hole pair density is concentrated. The distinguishing feature is that the isosurfaces are centrosymmetricly clustered on both sides of the molecular system and the area without isosurfaces in the intenal forms the axis of symmetry, see Figure S1g. When the twisted angle is 32.2°, TDM shows that the transition density distribution position of S_{49} has changed, see Figure S1d. The aggregation and distribution of the hole-electron isosurface has also changed at the same time, see Figure 2h. This is due to the change in the contribution of the atomic basis function of the MS based on the periodic complementary twisted angle to the transition dipole moment in the same edge region, which causes changes in orbital wavefunction, thereby the transition produces an antisymmetric electron-hole pair density.

The other excited state of the secondary peak is S_{53} at 598.1nm. Compared with S_{49}, the overlap and dispersion of electron holes are similar. Since the t index is less than 0, there is no significant separation of holes and electrons in the direction of charge transfer. However, the relative values are significantly different, indicating that the average ductility of electron- holes decreases after the twisted angle is changed from 27.8° to 32.2°. TDM shows that S_{53} belongs to local excitation, the hole-electron distribution is similar to S_{49}, and its isosurface distribution has an obvious axis of symmetry, see Figure S2(b and c) and Figure S2(e and f). The above discussion shows that the electronic excitation characteristics of Z-TwBLG-QDs based on periodic complementary twisted angles are mainly interlayer local excitation. The distinguishing feature of electron-hole pairs is the antisymmetric distribution.

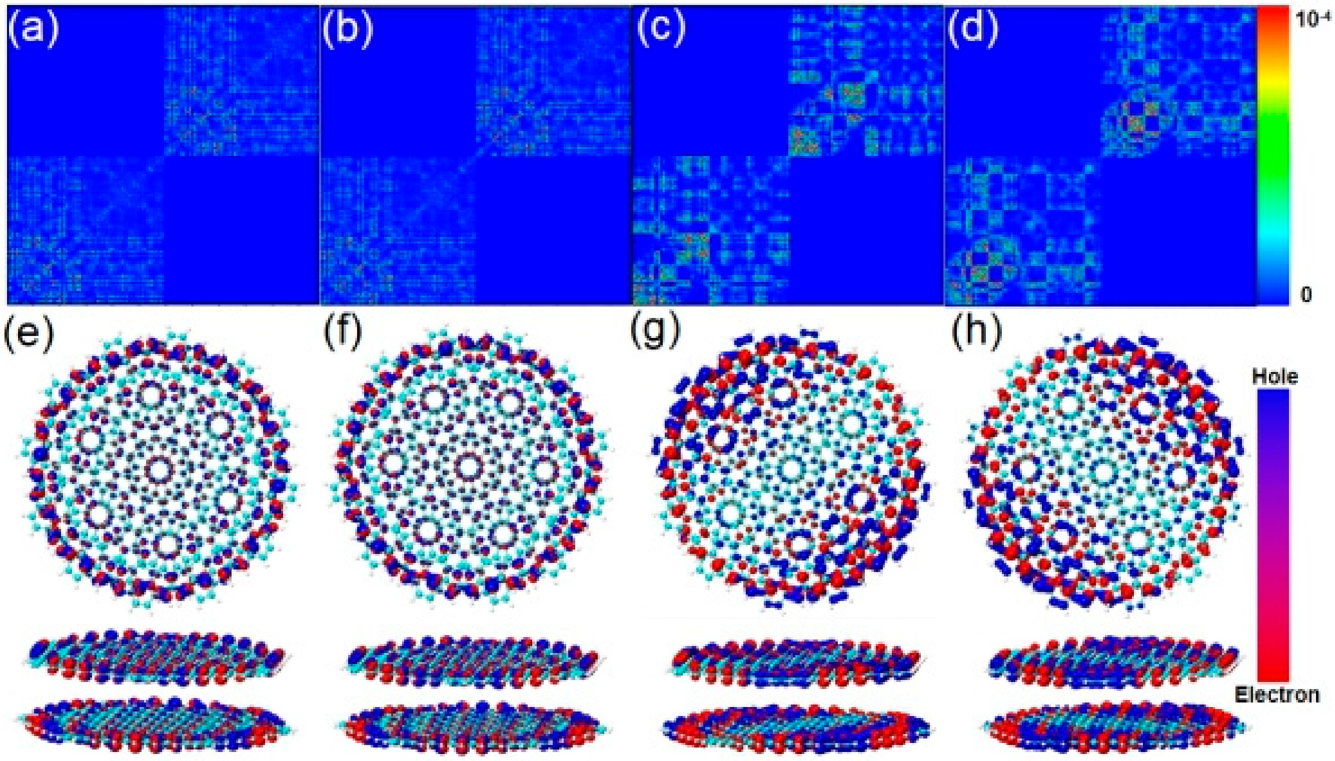

Figure S1. TDM and electron-hole density of Z-TwBLG-QDs-27.8° (a, e) and Z-TwBLG-QDs-32.2° at S_{15} (b, f), respectively. The TDM and electron-hole pair density of Z-TwBLG-QDs-27.8° (c, g) and Z-TwBLG-QDs-32.2° (d, h) at S_{49}, respectively.

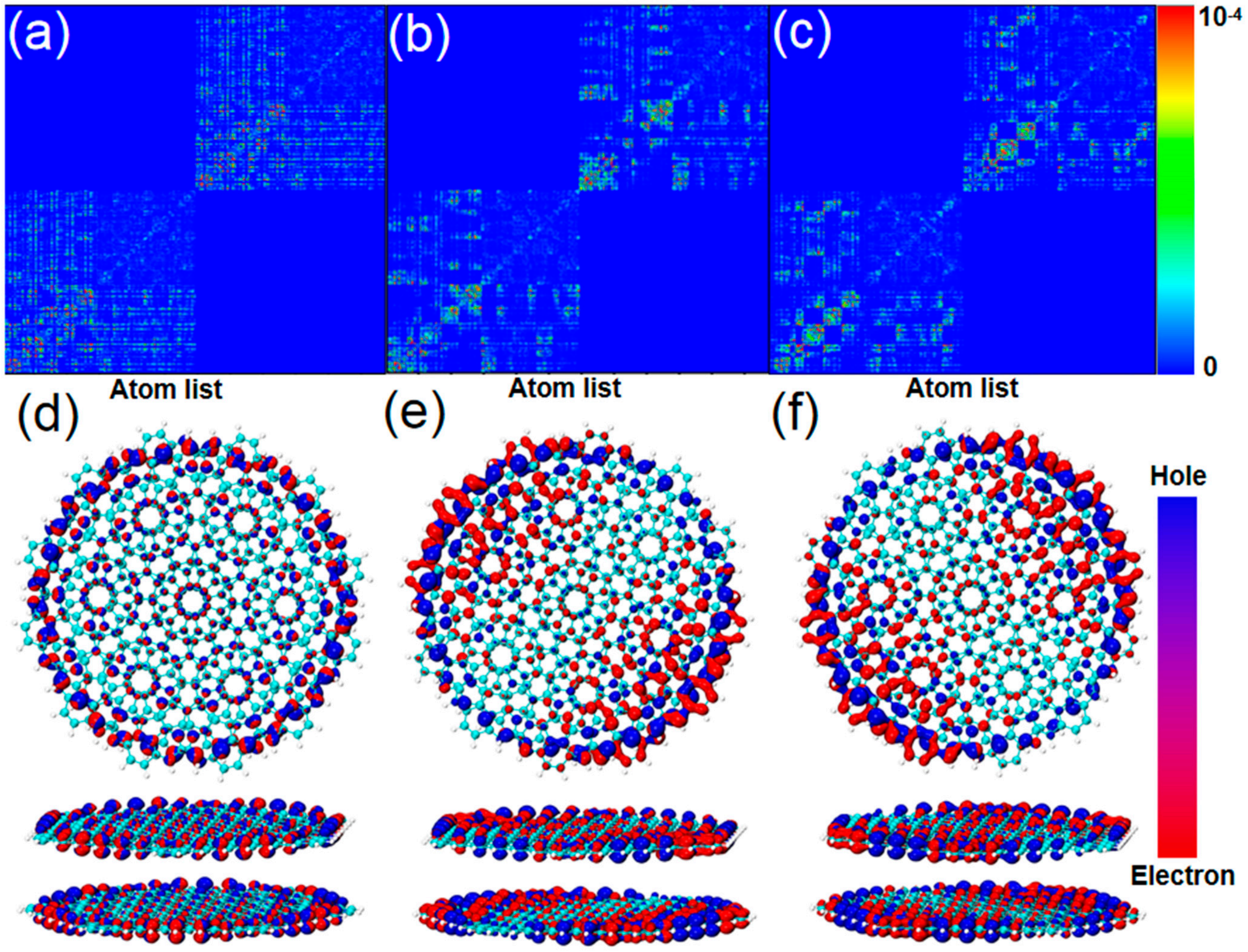

Figure S2. TDM and electron-hole pair density of Z-TwBLG-QDs-27.8° (a, d) at S_{16}. TDM and electron-hole pair density of Z-TwBLG-QDs-27.8° (b, e) and Z-TwBLG-QDs-32.2° (c, f) at S_{53}, respectively.

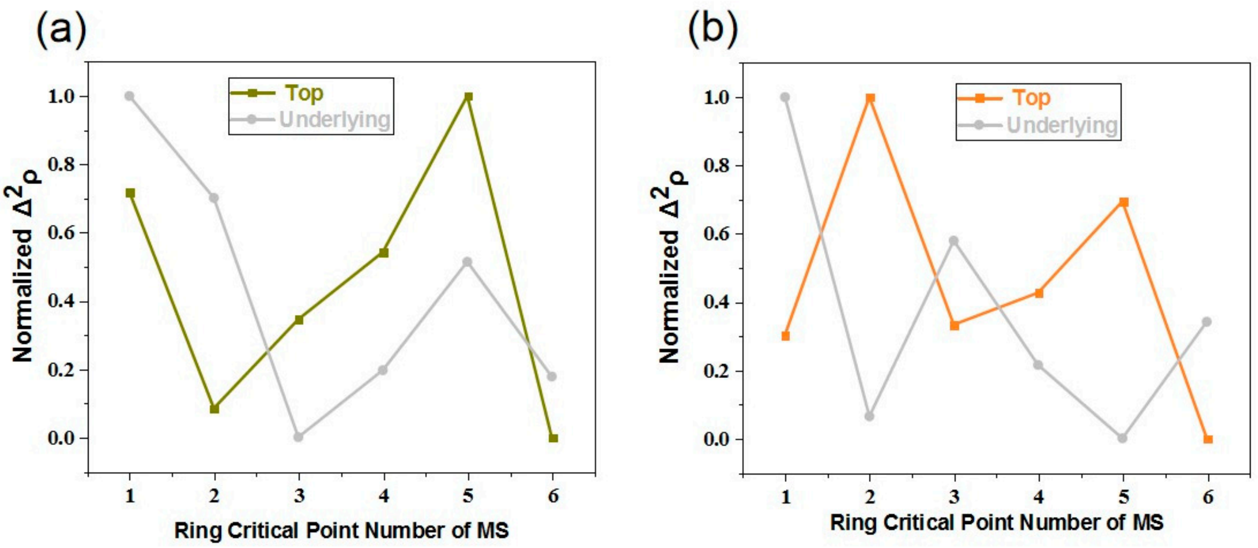

Figure S3. The normalized $\nabla^2_{\rho_{RCP}}$ of Z-TwBLG-QDs-27.8° and Z-TwBLG-QDs-32.2° (a, b)

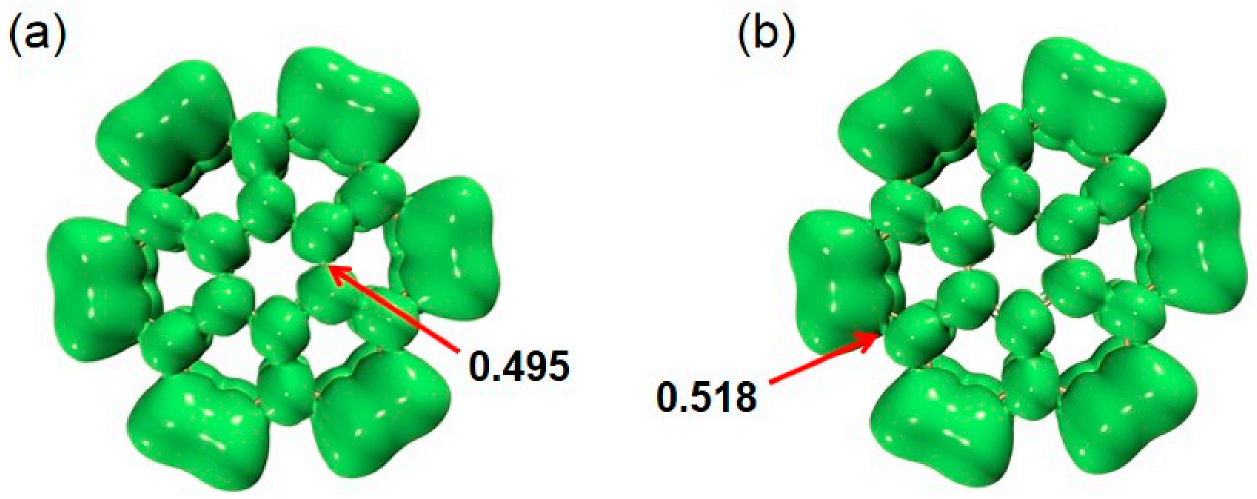

Figure S4. (**a**) The isovalue of the central six-membered ring of Coronene when the connected region of the ELF_pi isosurface was just fractured (0.495). (**b**) The isovalue of the edge of Coronene when the connected region of the ELF_pi isosurface was just fractured (0.518).

Table S4. The real space function values of the RCP in Coronene.

	RCP	ρ_{RCP}	G_{RCP}	K_{RCP}	$\nabla^2\rho_{RCP}$	$ELF_{(RCP)}$	LOL_{RCP}	ESP_{RCP}
Coro-nene	1	0.019416702	0.023931936	-0.00426603	0.112791870	0.027518094	0.144045734	31.52709441

Table S5. The real space function values of the RCP at the Moiré in top (T) and underlying (U) layer Z-TwBLG-QDs-27.8°.

	RCP	ρ_{RCP}	G_{RCP}	K_{RCP}	$\nabla^2\rho_{RCP}$	$ELF_{(RCP)}$	LOL_{RCP}	ESP_{RCP}
T	1	0.022894059	0.030871942	-0.006226336	0.148393113	0.028610835	0.146521651	174.5892277
	2	0.022894007	0.030871852	-0.006226323	0.148392704	0.028610788	0.146521544	174.5892835
	3	0.022894028	0.030871889	-0.006226329	0.148392870	0.028610807	0.146521587	174.5903556
	4	0.022894044	0.030871917	-0.006226333	0.148392997	0.028610822	0.146521621	174.5908577
	5	0.022894082	0.030871982	-0.006226342	0.148393295	0.028610856	0.146521698	174.5911243
	6	0.022894000	0.030871840	-0.006226322	0.148392648	0.028610781	0.146521529	174.5907359
	7	0.022965512	0.030994451	-0.006243284	0.148950940	0.028679530	0.146675927	191.4333295
U	1	0.022894046	0.030871946	-0.006226332	0.148393112	0.028610777	0.146521520	174.5901997
	2	0.022894028	0.030871914	-0.006226327	0.148392966	0.028610760	0.146521481	174.5901465
	3	0.022893985	0.030871839	-0.006226317	0.148392623	0.028610721	0.146521395	174.5907319
	4	0.022893997	0.030871860	-0.006226320	0.148392720	0.028610732	0.146521419	174.5901909
	5	0.022894016	0.030871894	-0.006226324	0.148392873	0.028610748	0.146521454	174.5899482
	6	0.022893996	0.030871858	-0.006226320	0.148392711	0.028610731	0.146521416	174.5903583
	7	0.022965518	0.030994489	-0.006243284	0.148951093	0.028679490	0.146675836	191.4333330

Table S6. The real space function values of the RCP at the Moiré in top (T) and underlying (U) layer of Z-TwBLG-QDs-32.2°.

	RCP	ρ_{RCP}	G_{RCP}	K_{RCP}	$\nabla^2 \rho_{RCP}$	ELF_{RCP}	LOL_{RCP}	ESP_{RCP}
	1	0.022893348	0.030870746	-0.006226158	0.148387618	0.028610114	0.146520029	174.5929661
	2	0.022893411	0.030870854	-0.006226174	0.148388111	0.028610171	0.146520158	174.5921391
	3	0.022893351	0.030870751	-0.006226159	0.148387640	0.028610116	0.146520053	174.5928747
T	4	0.022893359	0.030870765	-0.006226161	0.148387703	0.028610124	0.146520053	174.5938222
	5	0.022893383	0.030870807	-0.006226167	0.148387894	0.028610146	0.146520102	174.5940527
	6	0.022893321	0.030870699	-0.006226152	0.148387403	0.028610089	0.146519973	174.5942027
	7	0.022973551	0.031008469	-0.006245237	0.149014825	0.028686848	0.146692347	191.4394635
	1	0.022893412	0.030870831	-0.006226175	0.148388023	0.028610216	0.146520259	174.5934104
	2	0.022893361	0.030870745	-0.006226163	0.148387630	0.028610167	0.146520150	174.5931932
	3	0.022893388	0.308707923	-0.006226169	0.148387847	0.028610194	0.146520209	174.5935141
U	4	0.022893369	0.308707585	-0.006226164	0.148387692	0.028610176	0.146520169	174.5936698
	5	0.022893358	0.308707388	-0.006226162	0.148387602	0.028610164	0.146520143	174.593000
	6	0.022893376	0.308707706	-0.006226166	0.148387747	0.028610181	0.146520181	174.5932810
	7	0.022973550	0.031008440	-0.006245237	0.149014700	0.028686890	0.146692400	191.4395000
